# Association of BMI Category Change with TB Treatment Mortality in HIV-Positive Smear-Negative and Extrapulmonary TB Patients in Myanmar and Zimbabwe

**DOI:** 10.1371/journal.pone.0035948

**Published:** 2012-04-24

**Authors:** Lenka Benova, Katherine Fielding, Jane Greig, Bern-Thomas Nyang'wa, Esther Carrillo Casas, Marcio Silveira da Fonseca, Philipp du Cros

**Affiliations:** 1 London School of Hygiene and Tropical Medicine, London, United Kingdom; 2 Médecins Sans Frontières, London, United Kingdom; 3 Médecins Sans Frontières, Amsterdam, Holland; San Francisco General Hospital, University of California San Francisco, United States of America

## Abstract

**Objective:**

The HIV epidemic has increased the proportion of patients with smear-negative and extrapulmonary tuberculosis (TB) diagnoses, with related higher rates of poor TB treatment outcomes. Unlike in smear-positive pulmonary TB, no interim markers of TB treatment progress are systematically used to identify individuals most at risk of mortality. The objective of this study was to assess the association of body mass index (BMI) change at 1 month (±15 days) from TB treatment start with mortality among HIV-positive individuals with smear-negative and extrapulmonary TB.

**Methods and Findings:**

A retrospective cohort study of adult HIV-positive new TB patients in Médecins Sans Frontières (MSF) treatment programmes in Myanmar and Zimbabwe was conducted using Cox proportional hazards regression to estimate the association between BMI category change and mortality. A cohort of 1090 TB patients (605 smear-negative and 485 extrapulmonary) was followed during TB treatment with mortality rate of 28.9 per 100 person-years. In multivariable analyses, remaining severely underweight or moving to a lower BMI category increased mortality (adjusted hazard ratio 4.05, 95% confidence interval 2.77–5.91, p<0.001) compared with remaining in the same or moving to a higher BMI category.

**Conclusions:**

We found a strong association between BMI category change during the first month of TB treatment and mortality. BMI category change could be used to identify individuals most at risk of mortality during TB treatment among smear-negative and extrapulmonary patients.

## Introduction

In 2010, approximately 8.8 million incident cases of tuberculosis (TB) occurred, 1.45 million people died from the disease and 24% of those who died were co-infected with the human immunodeficiency virus (HIV) [1]. In countries with a high prevalence of HIV, HIV-positive individuals have a 20-fold increased risk of developing TB compared with those who are HIV-negative [2]. Antiretroviral therapy (ART) can substantially reduce TB incidence among HIV-positive patients [3]. However, TB slows immunological recovery among patients on ART [4], and mortality after initiation of TB treatment is higher among HIV-positive patients [5]–[10]. In South Africa, TB incidence rates remained two-fold among HIV-positive patients with CD4 cell counts >500/mm^3^, compared with HIV-negative individuals [6].

TB is more likely to be smear-negative and extrapulmonary in HIV-positive than in HIV-negative patients, irrespective of ART status [6], [11]. Overall, 63% of 5,930 adult HIV-positive TB patients with information on clinical presentation enrolled in TB treatment by Médecins Sans Frontières (MSF) in six countries between January 2006 and September 2008 were smear-negative or extrapulmonary [12]. These patients are at higher risk of TB treatment default and mortality, partly as a result of delayed diagnosis and treatment [7], [13], [14]. Both Zimbabwe and Myanmar are in the WHO ‘high TB burden’ category and most notified new TB cases in both countries (51% and 66%, respectively) are either smear-negative or extrapulmonary [15], [16].

WHO guidelines on interim indicators of treatment success in TB patients focus on smear-positive patients (i.e. cohort smear conversion rate of 80% in month 2 of TB treatment). No prognostic indicators are currently systematically applied among smear-negative and extrapulmonary TB patients to identify those at increased risk of unfavourable TB treatment outcomes. HIV-related malnutrition increases the risk of TB [17], [18] and is exacerbated by TB, with a negative overall impact on mortality [10], [19]–[21]. Among patients with HIV, those diagnosed with pulmonary TB have lower body mass index (BMI) and weight than those without TB [22]. In HIV patients without TB, higher BMI is associated with better survival outcomes [23] and low BMI is a predictor for mortality even in patients receiving ART [18].

If it is successful, TB treatment should result in weight gain among underweight individuals through restoring muscle and fat mass, depending on nutritional intake [19]. Previous studies on cohorts of mixed HIV status have shown that weight changes during early TB treatment can be useful indicators of TB treatment outcomes [24], [25]. However, the association of this indicator has not yet been assessed among smear-negative and extrapulmonary HIV-positive patients.

In this study, we aimed to establish how changes in BMI category after the first month of TB treatment in new adult smear-negative and extrapulmonary patients co-infected with HIV were associated with mortality during TB treatment.

## Materials and Methods

Routinely collected patient data from three MSF TB treatment programmes (Shan and Yangon in Myanmar, Gweru in Zimbabwe) were used in this retrospective cohort study. Data from electronic HIV and TB databases were merged and all HIV-positive patients starting treatment for smear-negative or extrapulmonary tuberculosis between February 6, 2003 and July 17, 2010 were identified. Smear-negative and extrapulmonary TB were diagnosed according to the recommended WHO algorithm [26]. Acid-fast bacilli smear tests were available for diagnosis; no cultures were performed. No diagnosis or treatment for drug-resistant TB was available.

Inclusion was limited to new, adult TB patients, defined as patients who had not taken TB drugs for more than 30 days within the five years preceding current TB treatment and who were ≥15 years at the start of TB treatment. To enable measurement of BMI category change between the start and one month of TB treatment, only patients followed at least until day 15 of TB treatment were considered.

**Figure 1:**
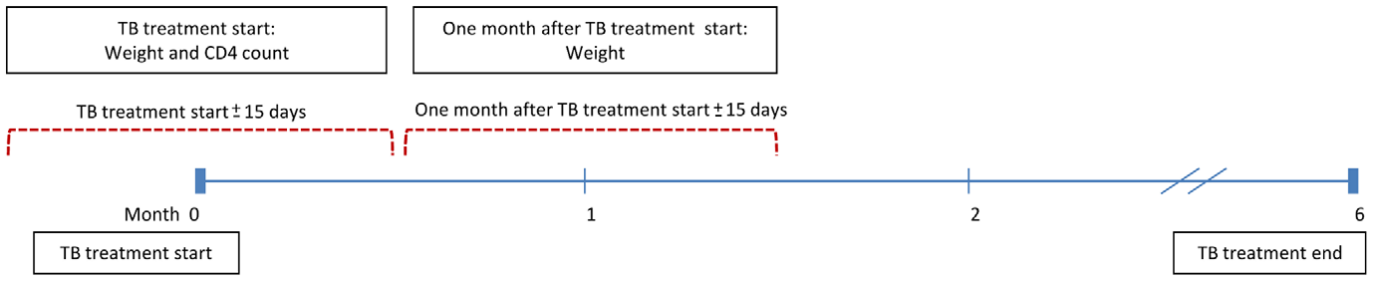
Intervals for measurement of weight and CD4 count at TB treatment start and 1 month after TB treatment start.

Patients without a recorded TB treatment outcome were excluded (e.g. those who transferred to another healthcare provider during treatment). Further, only patients with a weight measurement at the start of TB treatment (within ±15 days of treatment start date) and one month after TB treatment start (between 15 and 45 days from treatment start date), as well as a height measurement anytime during TB treatment, were included (Figure 1).

Our main outcome measure was all-cause mortality during TB treatment and our secondary outcome was unfavourable TB treatment outcome (combination of death, default and treatment failure). The binary exposure, change in BMI category between the start and one month of TB treatment, was defined as remaining severely underweight or moving to a lower BMI category versus remaining in the same or moving to a higher BMI category. BMI was calculated by dividing the weight in kilograms by square of height in meters and categorized according to standard definitions (severely underweight: <16; underweight 16.00–18.49; normal 18.5–24.99; overweight and obese ≥25, kg/m^2^).

MSF guidelines on the timing of initiation of ART in HIV-positive TB patients changed during the study. In Myanmar, before June 2007, all TB patients fulfilled criteria to start ART; from June 2007 to late 2009, all extrapulmonary TB (irrespective of CD4 count) and pulmonary TB patients with CD4<350/mm^3^ qualified for ART; and from January 2010, all TB cases started ART irrespective of CD4 count. In Zimbabwe, from 2007 to 2010, all patients with WHO stage 4 and patients with stage 3 with CD4 count<350/mm^3^ started ART. Prior to 2010, if CD4 count was <200/mm^3^, ART was started within the intensive phase of TB treatment; if it was ≥200/mm^3^ it was started upon completion of the intensive phase. As of 2010, TB patients were initiated on ART regardless of CD4 count as soon as possible after TB treatment initiation.

Data were analyzed using Stata/SE v.12 (Stata Corporation, College Station, Texas, USA). Follow-up was measured from start of TB treatment and ended either on the date TB treatment was finished (if patients completed treatment); on the date TB treatment was stopped (if patients failed or defaulted from treatment), or on the recorded date of death for those who died during TB treatment.

Cox proportional hazards regression was used to obtain unadjusted hazard ratios for the effect of BMI category change on time to death. Each potential confounder was added and evaluated by its strength in meaningfully adjusting the modelled hazard ratio for BMI category change. In patients not receiving ART prior to TB treatment, exposure to ART is likely to change over the course of TB treatment as they become eligible for ART. Therefore, ART status was modelled as a binary time-dependent covariate. This approach allows for person-time at risk in both categories of ART status to be analyzed more precisely. Project site, exposure to ART at the start of TB treatment and sex were considered *a priori* effect modifiers and assessed in final models using the likelihood ratio test. Each adjusted Cox proportional hazards model was assessed to confirm proportionality of hazard functions with BMI category change. Analysis of the effect of BMI change on the composite endpoint of unfavourable TB treatment outcome was conducted using the same modelling approach. In addition, a sensitivity analysis was conducted on a larger sample of patients (n = 1330) surviving up to day 30 of TB treatment, where change of BMI category between the start and 2 months of TB treatment (weight measurements interval ±30 days) was assessed.

This study was approved by the Research Ethics Committee of the London School of Hygiene and Tropical Medicine, UK and met the standards set by the MSF Ethics Review Board for retrospective analysis of routinely collected programmatic data.

## Results

**Figure 2:**
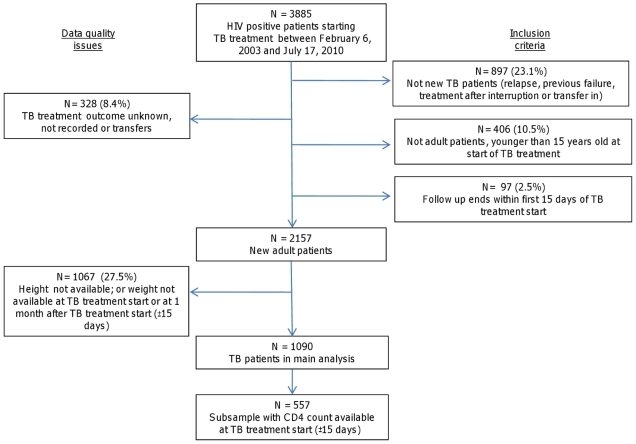
Flow diagram showing sample construction.

The final sample consisted of 1090 TB patients (Figure 2), 605 and 485 with smear-negative and extrapulmonary TB, respectively. Among this sample, 34% were from Shan, 29% from Yangon and 37% from Gweru (Table 1). The median age was 34 years and overall 54% were male. More than 60% of patients were underweight or severely underweight at the start of TB treatment. Whereas only 610 (56%) of the overall patient sample had a record for cotrimoxazole preventive therapy (CPT) status, more than 97% of the patients with a non-missing value received CPT. The total follow up time was 519.4 person-years and the median follow up time was 182 days. A total of 150 deaths occurred during TB treatment (14% of sample), giving an overall mortality rate of 28.9 per 100 person-years (95% confidence interval [CI] 24.6–33.9). The smear-negative and extrapulmonary patient samples have similar distributions of demographic and baseline epidemiological variables, with the exception of the proportion starting ART before TB treatment start and TB treatment end (Table 2).

**Table 1:** Characteristics of final TB patient sample at TB treatment start, ART exposure and TB treatment duration.

	Overall		Project site	
		Shan	Yangon	Gweru
n	1090	369	317	404
TB type				
Smear-negative (%)	55.5	53.4	52.7	59.7
Extrapulmonary	44.5	46.6	47.3	40.3
Sex				
Male (%)	53.9	64.0	59.9	39.9
Age (years)				
Median	34.0	33.2	32.3	36.3
IQR	29.5–40.2	29.9–39.1	28.1–38.1	30.8–43.5
Weight at TB treatment start (kg)				
Median	47	45	44	51
IQR	41–53	39–51	39.5–50	45–57
BMI category at TB treatment start (kg/m^2^)				
<16 (%)	23.6	27.9	27.4	16.6
16–18.49	36.8	41.7	38.5	30.9
18.5–24.99	36.8	29.3	32.2	47.3
≥25	2.8	1.1	1.9	5.2
TB treatment regimen				
2RHEZ/4RH (%)*	98.4	99.4	99.5	96.5
ART status at TB treatment start				
Prior to TB treatment (%)	21.3	22.2	16.1	24.5
Within 2 weeks of TB treatment	8.7	5.4	15.8	6.2
Within 2–4 weeks of TB treatment	18.0	18.2	22.1	14.6
After 4 weeks of TB treatment	31.9	26.0	34.0	35.6
After end of TB treatment	20.1	28.2	12.0	19.1
TB treatment duration (days)				
Median	182	180	179	184
IQR	169–192	164–192	169–190	181–196
Range	20–349	20–349	34–329	21–294
*Subsamples with non-missing values*				
CPT before TB treatment start				
n	610	123	84	403
Yes (%)	97.4	99.2	95.2	97.3
CD4 count at TB treatment start (/mm^3^)				
n	557	207	157	193
Median	82	70	95	84
≤100 (%)	57.8	64.2	50.3	57.0
101–200	21.5	15.5	31.9	19.7
201–350	14.2	14.0	14.0	14.5
>350	6.5	6.3	3.8	8.8
Haemoglobin at TB treatment start (g/dL)				
n	434	116	119	199
Median	9.5	9.8	9.5	9.3
≤8.5 (%)	35.0	38.8	28.6	36.7

* 2 months of rifampicin (R), isoniazid (H), pyrazinamide (Z), ethambutol (E), followed by 4 months of rifampicin (R) and isoniazid (H). Patients not on this recommended regimen were either receiving different treatment due to side effects, or were receiving streptomycin instead of ethambutol with a diagnosis of extrapulmonary meningitis.

IQR = interquartile range. ART = antiretroviral therapy. BMI = body mass index. CPT = cotrimoxazole preventive therapy. TB = tuberculosis.

**Table 2:** Association of smear-negative and extrapulmonary TB patient samples with basic demographic and epidemiologic variables.

	Smear-negative	Extrapulmonary	Chi-square p-value
n	605	485	
Sex			
Male (%)	54.4	53.2	0.697
Age group			
15–24 (%)	6.1	8.0	
25–34	45.8	50.9	
35–44	31.1	29.1	0.072
45–54	13.0	8.5	
55 and above	4.0	3.5	
Project site			
Shan (%)	32.6	35.5	
Yangon	27.6	30.9	0.105
Gweru	39.8	33.6	
ART before TB treatment start			
Yes (%)	16.5	27.2	<0.001
ART before TB treatment end			
Yes (%)	82.3	76.9	0.027
BMI category at TB treatment start (kg/m^2^)			
<16 (%)	24.1	22.9	
16–18.49	37.4	36.1	0.069
18.5–24.99	36.9	36.7	
≥25	1.6	4.3	
*Subsamples with non-missing values*			
CD4 count at TB treatment start (/mm^3^)			
n	316	241	
≤100 (%)	61.7	52.7	
101–200	20.6	22.8	0.099
201–350	11.4	17.8	
>350	6.3	6.7	

ART = antiretroviral therapy. TB = tuberculosis.

A total of 81 deaths and 126 unfavorable outcomes were recorded among the 605 smear negative TB patients, compared to 69 deaths and 120 unfavorable outcomes among the sample of 485 extrapulmonary individuals. The log rank test for equality of survivor functions shows that the data are consistent with no difference in survivor functions for mortality or unfavorable treatment outcomes between the smear-negative and extrapulmonary TB patient samples (p = 0.908 and 0.451, respectively).

During the first month of treatment 27% (299/1090) of TB patients remained severely underweight or moved to a lower BMI category. The mortality rate among patients in this group was 61.4 per 100 person-years (95%CI 49.3–76.5), compared with 17.9 (95%CI 14.2–22.7) among those who remained in the same BMI category or gained a BMI category. Crude analysis showed strong evidence that the hazard for TB treatment mortality differed by category of BMI change and this association strengthened when adjusted for sex, age group, project, ART status and BMI at TB treatment start (Table 3). Remaining severely underweight or moving to a lower BMI category had 4.05 times (95%CI 2.77–5.91, p<0.001) higher mortality rate compared with remaining in the same or moving to a higher BMI category. Data were consistent with no interaction of BMI category change with ART status at TB treatment start (p = 0.32), project site (p = 0.28) or sex (p = 0.64) and satisfied the assumption of proportional hazards (p = 0.16). Adjusting for CD4 count measured at the start of TB treatment showed a stronger magnitude of association between BMI category change and TB treatment mortality (Table 3).

**Table 3:** Univariable and multivariable association of BMI category change with TB treatment mortality.

Main sample (n = 1090)			Crude			Adjusted #		
	n	Number of deaths	HR	95% CI	p-value*	HR	95% CI	p-value*
Remained severely underweight or lost a BMI category	299	80	3.44	2.50–4.75	<0.001	4.05	2.77–5.91	<0.001
Stable or increased BMI category	791	70	1			1		

Sub-sample (n = 557)			Crude			Adjusted ##		
Patients with CD4 count at TB treatment start	n	Number of deaths	HR	95% CI	p-value*	HR	95% CI	p-value*
Remained severely underweight or lost a BMI category	168	50	4.24	2.71–6.64	<0.001	5.25	3.09–8.91	<0.001
Stable or increased BMI category	389	31	1			1		

* p-value of likelihood ratio test.

# Adjusted for sex, age group, project, ART as time dependent variable and BMI category at TB treatment start.

## Adjusted for sex, age group, project, ART as time dependent variable, BMI category and CD4 category at TB treatment start.

CI = confidence interval. HR = hazard ratio. TB = tuberculosis. ART = antiretroviral therapy. BMI = body mass index.

Sensitivity analysis on a sample of 1330 patients surviving up to day 30 of TB treatment showed that the direction and magnitude of the association between BMI category change and TB treatment mortality was very similar to the main model presented (Appendix S1). Lastly, those who remained severely underweight or lost a BMI category had over twice the rate of unfavourable TB treatment outcomes compared to those who remained in the same or moved to a higher BMI category (adjusted HR 2.53, 95%CI 1.87–3.42; Appendix S2).

## Discussion

Our results show a strong association between BMI category change during early TB treatment and TB treatment outcomes (mortality and combined unfavourable outcomes) among HIV-positive TB smear-negative and extrapulmonary individuals. Our sizeable study sample included TB patients from three clinical programmes in two countries.

In HIV-positive new adult smear-negative and extrapulmonary TB patients, after adjustment, remaining severely underweight or losing a BMI category in the first month of TB treatment increased mortality four-fold compared with remaining in the same or moving to a higher BMI category. The association was even stronger in a subset of patients after an adjustment was made for CD4 count at TB treatment start. There is limited guidance on systematic evaluation of increased risk of death during TB treatment among HIV-positive smear-negative and extrapulmonary TB patients. Our study shows that a simple binary change in BMI category during the first month of TB treatment, an inexpensive measure that is collected and recorded during routine clinical visits, could be used for interim monitoring of these patients.

Previous studies have reported that weight gain is a useful interim marker for favourable TB outcomes in smear-positive TB patients. Among pulmonary smear-positive patients, unfavourable TB treatment outcomes were associated with body weight gain of ≤5% at the end of TB treatment, although not at one month after TB treatment start [27]. Further, among new adult TB patients (pulmonary smear-positive and negative, and extrapulmonary), weight gain of almost 1 kg after one month on TB treatment was found to be strongly associated with favourable TB treatment outcomes. The study also found that most of the change in body weight differentiating individuals with favourable versus unfavourable TB treatment outcomes happened during the first month of TB treatment and body weight change was found to be an independent predictor of outcome to interim smear microscopy conversion [24]. However, both these studies had low numbers of patients with known HIV status and used weight change (absolute or relative) as the exposure, a more cumbersome measure than change in standard BMI categories.

The limitations of this study pertain to the nature of the data, collected during routine clinical practice. Patients starting TB treatment over a period of seven years were included, and diagnostic and treatment guidelines, not only for TB and ART, but also for malnutrition and HIV opportunistic illnesses, changed during this time. In order to control for ART status, an important confounder, it was adjusted for as a time-dependent covariate. This study was conducted with data collected before greater access to rapid molecular diagnostics became available in the programme areas, thus patients were diagnosed according to clinical algorithms which included clinical assessment, sputum microscopy and chest radiography based on WHO guidelines [28]. The lack of mycobacterial culture for diagnosis means that it is likely that not all patients actually had TB. In the absence of culture and drug sensitivity testing this study focused on new TB patients. It is also possible that there were some cases of drug-resistant TB. However, such numbers are likely to be small given that the prevalence of multi-drug-resistant TB among new patients in 2008 was estimated at 1.9% in Zimbabwe and 4.2% in Myanmar [15], [16].

Further, data on occurrence of immune reconstitution inflammatory syndrome (IRIS) was not systematically available in this routine programmatic database, and was therefore not included in the analysis. TB IRIS is very common and could potentially have contributed to reduction or lack of change in BMI. However, as our primary outcome measure is mortality during TB treatment, and BMI category is a simple interim measure that can be included in routine programme monitoring, the lack of data on TB IRIS does not change the interpretation of our findings. Previous studies have shown that a proportion of TB treatment defaulters (8.4% of our initial sample) may in fact have died [29], and thus we may have underestimated the number of TB treatment deaths. Finally, individuals with height and weight measurements available were somewhat different from patients without these measurements, particularly in regards to distribution by project and ART status, although their survivor functions do not differ (Appendix S3).

In summary, our findings reveal that remaining severely underweight or losing a BMI category during the first month of TB treatment can predict treatment outcome among new adult HIV-positive individuals with smear-negative and extrapulmonary TB. Currently, there are no interim programme monitoring indicators for this group of patients, reported to have the highest risk of mortality and unfavourable outcomes in countries with high prevalence of HIV and TB [7], [13], [14], [30]–[33]. There is an urgent need for interim monitoring of both individual patients and patient cohorts in areas with a high prevalence of HIV and TB. BMI change monitoring is a simple and inexpensive tool that is feasible to implement under routine programmatic conditions in resource-limited settings. Patients identified as being at high risk could benefit from more intensive monitoring, nutritional supplementation and be prioritized for further investigations, such as culture and multi-drug-resistant TB testing, which may not be feasible for all TB patients in such settings. Further research is needed to evaluate whether the use of this interim marker can help improve patient outcomes through such earlier and more efficient use of patient monitoring, investigations and support.

## Supporting Information

Appendix S1 Univariable and multivariable association of BMI category change between TB treatment start and 2 months after TB treatment start with TB treatment mortality on a sample of patients surviving up to day 30 of TB treatment.
(DOCX)

Appendix S2 Univariable and multivariable association of BMI category change with unfavourable TB treatment outcome (default, failure, death).
(DOCX)

Appendix S3 Association of two patient samples with TB type, sex, age group, project site and ART status at TB treatment start.
(DOCX)
