# Supplementary material for: Association of BMI Category Change with TB Treatment Mortality in HIV-Positive Smear-Negative and Extrapulmonary TB Patients in Myanmar and Zimbabwe
Source: PLoS One. 2012 Apr 24;7(4):e35948. doi: 10.1371/journal.pone.0035948 (PMC3335812; doi:10.1371/journal.pone.0035948)
Supplement: Appendix S1 — Univariable and multivariable association of BMI category change between TB treatment start and 2 months after TB treatment start with TB treatment mortality on a sample of patients surviving up to day 30 of TB treatment. (DOCX) [file pone.0035948.s001.docx]

**Appendix S1.**

| **Final sample (n=1330)** |  | |  | Crude | | | Adjusted # | | |
| --- | --- | --- | --- | --- | --- | --- | --- | --- | --- |
|  | n | | Number of deaths | HR | 95% CI | p-value* | HR | 95% CI | p-value* |
| Remained severely underweight or lost a BMI category | 306 | | 75 | 4.20 | 3.02-5.85 | <0.001 | 4.80 | 3.27-7.05 | <0.001 |
| Stable or higher BMI category | 1024 | | 67 | 1 |  |  | 1 |  |  |
| **Sub-sample (n=903)** |  | |  | Crude | | | Adjusted ## | | |
| **Patients with CD4 count at TB treatment start** | | n | Number of deaths | HR | 95% CI | p-value* | HR | 95% CI | p-value* |
| Remained severely underweight or lost a BMI category | | 214 | 57 | 4.78 | 3.22-7.09 | <0.001 | 5.16 | 3.24-8.21 | <0.001 |
| Stable or higher BMI category | | 689 | 44 | 1 |  |  | 1 |  |  |

*p-value of likelihood ratio test.

#Adjusted for sex, age group, project, ART as time dependent variable and BMI category at TB treatment start.

## Adjusted for sex, age group, project, ART as time dependent variable, BMI category and CD4 category at TB treatment start.

CI=confidence interval. HR=hazard ratio. TB=tuberculosis. ART=antiretroviral therapy. BMI=body mass index.
