# Supplementary material for: Association of BMI Category Change with TB Treatment Mortality in HIV-Positive Smear-Negative and Extrapulmonary TB Patients in Myanmar and Zimbabwe
Source: PLoS One. 2012 Apr 24;7(4):e35948. doi: 10.1371/journal.pone.0035948 (PMC3335812; doi:10.1371/journal.pone.0035948)
Supplement: Appendix S2 — Univariable and multivariable association of BMI category change with unfavourable TB treatment outcome (default, failure, death). (DOCX) [file pone.0035948.s002.docx]

**Appendix S2.**

| **Main sample (n=1090)** |  |  | Crude | | | Adjusted # | | |
| --- | --- | --- | --- | --- | --- | --- | --- | --- |
|  | n | Number of events | HR | 95% CI | p-value* | HR | 95% CI | p-value* |
| Remained severely underweight or lost a BMI category | 299 | 102 | 2.17 | 1.69-2.80 | <0.001 | 2.53 | 1.87-3.42 | <0.001 |
| Stable or higher BMI category | 791 | 144 | 1 |  |  | 1 |  |  |
| **Sub-sample (n=557)** |  |  | Crude | | | Adjusted # | | |
| **Patients with CD4 count at TB treatment start** | n | Number of events | HR | 95% CI | p-value* | HR | 95% CI | p-value* |
| Remained severely underweight or lost a BMI category | 168 | 61 | 2.60 | 1.83-3.71 | <0.001 | 3.25 | 2.13-4.94 | <0.001 |
| Stable or higher BMI category | 389 | 62 | 1 |  |  | 1 |  |  |

*p-value of likelihood ratio test.

#Adjusted for sex, age group, project, ART as time dependent variable and BMI category at TB treatment start.

## Adjusted for sex, age group, project, ART as time dependent variable, BMI category and CD4 category at TB treatment start.

CI=confidence interval. HR=hazard ratio. TB=tuberculosis. ART=antiretroviral therapy. BMI=body mass index.
