# Supplementary material for: Association of BMI Category Change with TB Treatment Mortality in HIV-Positive Smear-Negative and Extrapulmonary TB Patients in Myanmar and Zimbabwe
Source: PLoS One. 2012 Apr 24;7(4):e35948. doi: 10.1371/journal.pone.0035948 (PMC3335812; doi:10.1371/journal.pone.0035948)
Supplement: Appendix S3 — Association of two patient samples with TB type, sex, age group, project site and ART status at TB treatment start. (DOCX) [file pone.0035948.s003.docx]

**Appendix S3.**

|  | Final sample | Patient records not used* | Chi-square p-value |
| --- | --- | --- | --- |
| n | 1090 | 1067 |  |
| TB type |  |  |  |
| Smear-negative (%) | 55.5 | 51.5 | 0.059 |
| Extrapulmonary | 44.5 | 48.5 |  |
| Sex |  |  |  |
| Male (%) | 53.9 | 54.3 | 0.848 |
| Age group |  |  |  |
| 15-24 (%) | 7.0 | 10.6 |  |
| 25-34 | 48.1 | 44.7 |  |
| 35-44 | 30.2 | 30.5 | 0.048 |
| 45-54 | 11.0 | 10.4 |  |
| 55 and above | 3.7 | 3.8 |  |
| Project site |  |  |  |
| Shan (%) | 37.1 | 46.4 |  |
| Yangon | 33.8 | 49.9 | <0.001 |
| Gweru | 29.1 | 3.7 |  |
| ART before TB treatment start |  |  |  |
| Yes (%) | 79.9 | 61.3 | <0.001 |

*Records not used due to weight measures not being available at the start of TB treatment (day 1 of TB treatment ±15 days) and/or at 1 month after TB treatment start (day 30 of TB treatment ±15 days) or height measurement not available anytime during TB treatment. ART=antiretroviral therapy. TB=tuberculosis.

A total of 196 deaths were recorded among the 1067 individuals without height and/or weight measurements. The log rank test for equality of survivor functions shows that the data are consistent with no difference in survivor functions between the final sample used for analysis and the group of 1067 individuals excluded (p=0.14).
